# Fast, Simple, and Sensitive Voltammetric Measurements of Acyclovir in Real Samples via Boron-Doped Diamond Electrode

**DOI:** 10.3390/ma17184480

**Published:** 2024-09-12

**Authors:** Damian Gorylewski, Katarzyna Tyszczuk-Rotko, Magdalena Wójciak, Ireneusz Sowa

**Affiliations:** 1 Faculty of Chemistry, Institute of Chemical Sciences, Maria Curie-Skłodowska University in Lublin, 20-031 Lublin, Poland; damian.gorylewski@mail.umcs.pl; 2 Department of Analytical Chemistry, Medical University of Lublin, 20-093 Lublin, Poland; magdalena.wojciak@umlub.pl (M.W.); ireneusz.sowa@umlub.pl (I.S.)

**Keywords:** acyclovir, herpes simplex virus, voltammetry, boron-doped diamond electrode, trace analysis

## Abstract

The voltammetric acyclovir (ACV) trace-level determination procedure has been introduced. This is the first time that a commercially available boron-doped diamond electrode (BDDE) coupled with differential-pulse voltammetry (DPV) has been used for this purpose. The commercially available BDDE is characterized by a short response time, low background current, and very good analytical parameters of ACV determination. Ultimately, DPV measurements using the BDDE in 0.075 mol L^−1^ PBS with a pH of 7.2 under optimized conditions achieved the lowest detection limit (LOD = 0.0299 nmol L^−1^) reported in the literature for voltammetric procedures. Moreover, it is highly resistant to the presence of various interfering agents and has been used to analyze pharmaceutical and municipal wastewater samples. The obtained results are consistent with measurements made using chromatographic reference methods.

## 1. Introduction

Compounds such as acyclovir (ACV), ganciclovir (GCV), and penciclovir (PCV) are commonly used in the treatment of the herpes simplex virus. Their action consists in inhibiting the replication of the virus, thanks to which the infection does not progress and the symptoms start to subside. This allows for shortened subsequent convalescence and minimizes the possibility of infecting other people [1,2,3].

**Figure 1:**
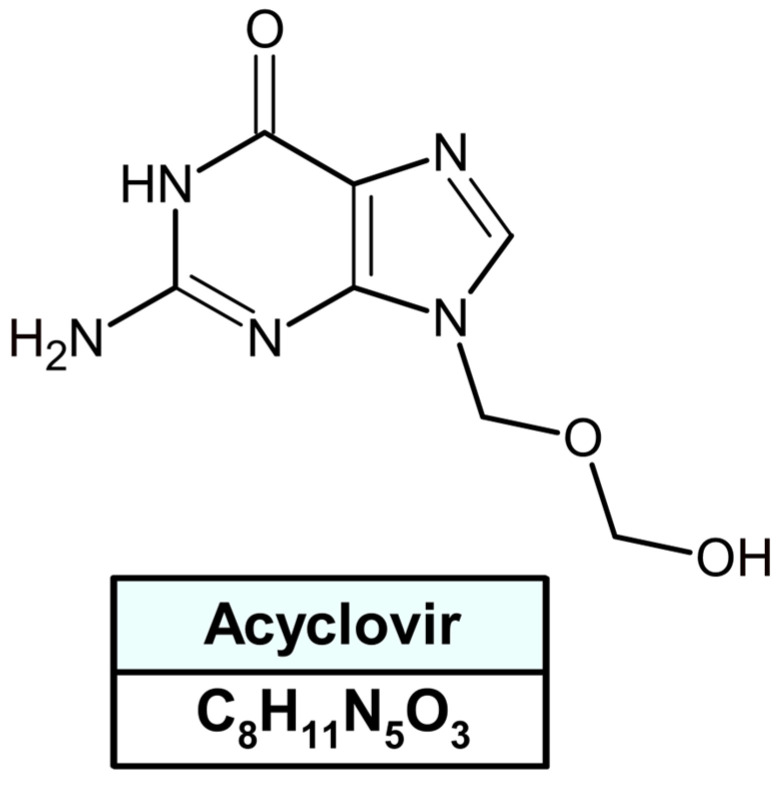
ACV structural formula.

ACV (2-amino-9-[(2-hydroxyethoxy)methyl]-1,9-dihydro-6H-purin-6-one, Figure 1) is used in the treatment of herpes simplex virus, human herpes virus 6, hepatitis B virus, Epstein–Barr virus, and varicella zoster viruses. It is one of the safest registered antiviral drugs that can be administered intravenously, orally, and topically with minimal risk of side effects. However, excessive consumption of ACV may lead to neurotoxicity, headaches, exacerbation of renal failure, cephalalgia, and diarrhea. ACV has a short life span in the human body and is only 15–20% metabolized. The other 80–85% of the consumed dose is excreted from the body unchanged mainly through the urine. In the treatment of herpes simplex virus, quite high doses of ACV are usually used. For example, for the treatment of genital herpes, ACV dosages for adults and children 12 years of age and older include 200 mg five times a day for 10 days, whereas the American Academy of Pediatrics recommends that high-dose (HD) acyclovir (20 mg kg^−1^/dose) should be used for the treatment of neonatal HSV disease [4]. Therefore, relatively large amounts of these compounds can end up in sewage and aquatic environments [5,6,7,8,9].

In the natural environment, ACV generally does not occur at high concentration levels, but the highest concentrations of acyclovir have been detected in wastewater treatment plants (WWTPs) (6.04 nmol L^−1^) and surface waters (7.06 nmol L^−1^). Additionally, ACV is inherently biodegradable in some amounts. There are indications that some ACV derivatives in high concentrations may be harmful to living organisms such as green algae or Daphnia magna [9]. The long-term effects of exposure to this compound in small amounts are currently unknown. It is very significant to develop effective monitoring tools for determining low concentrations of ACV in the environment.

Many procedures for the determination of ACV using spectroscopy [10], spectrophotometry [11,12], chemiluminescence [13], chromatography [14,15,16], or voltammetry [1,2,5,6,7,8,17,18,19,20,21,22,23,24,25,26,27,28,29,30,31,32,33,34,35,36,37,38,39,40] have been described in the literature (Table 1). Voltammetric procedures have several advantages over other instrumental methods. They are characterized by high sensitivity, low cost of apparatus, and low consumption of reagents and samples as well as they give the ability to perform measurements in field analysis conditions. Additionally, voltammetric methods allow the determination of metal ions and organic compounds to be conducted at trace concentration levels, without costly and time-consuming sample preparation [41,42]. Sample preparation in voltammetry is very cheap and mainly focuses on limiting interference, e.g., from organic substances that may be adsorbed on the electrode surface. Usually, performing a simple and fast filtration step or/and UV mineralization is sufficient to prepare the sample for most voltammetric analysis.

The vast majority of the ACV determination voltammetric procedures described in the literature are based on the use of difficult-to-prepare sensors such as the paste electrodes [20,23,27,30,33,43], gold electrodes (GE) [25], pencil graphite electrodes (PGE) [37,39], electropretrated pencil graphite electrodes (EPPGE) [26], graphite sheet electrodes (GSE) [32], screen-printed electrodes [44], or glassy carbon electrodes (GCE) [1,2,6,7,8,18,21,22,24,28,29,34,35,36,40]. They are primarily modified with, e.g., multi-walled nanotubes (MWNTs), graphene, fullerenes, polymers, nanodiamonds, nanoclay, and magnetic nanoparticles as well as being electrochemically activated. Unmodified electrodes such as GCE [5], pencil graphite electrode (PGE) [19], ultra-trace graphite electrode (UTGE) [5], fluoride doped oxide (FTO) [17], electropretreated pencil graphite electrode (EPPEG) [31], and controlled growth mercury drop electrode (CGME) [38] have also been applied in the analysis of ACV. However, the detection limits (LOD) that have been achieved with unmodified electrodes are quite high compared to the modified ones (Table 1).

In this paper, we would like to introduce the first application of the bare BDDE in ACV trace-level determination. The choice of this type of sensor was dictated by its properties, i.e., a short response time, high stability, chemical inertness, low background current, and good analytical parameters [45,46,47,48,49,50,51,52,53,54,55]. To optimize the ACV determination procedure and to characterize the properties of the sensor and electrode process, many experiments were carried out using several techniques, including differential-pulse voltammetry (DPV), square-wave voltammetry (SWV), and cyclic voltammetry (CV). Ultimately, the optimization of the procedure parameters, investigation of the influence of possible interfering agents, and the ACV analysis in environmental samples were done using DPV. The correctness of the obtained results was verified by performing real sample analysis using reference-chromatographic methods.

## 2. Materials and Methods

### 2.1. Equipment

The potentiostat/galvanostat (Eco Chemie, Utrecht, The Netherlands) with GPES 4.9 and FRA 4.9 software was applied to perform voltammetric and EIS analysis, respectively. Experiments were made in a 10 mL cell consisting of the commercially available BDDE (D-064-SA, boron doping level 1000 ppm, Windsor Scientific, Berkshire, UK) or GCE (glassy carbon electrode, Mineral, Łomianki-Sadowa, Poland) with a diameter of 3 mm (working electrodes), an Ag,AgCl/KCl (3 mol L^−1^) silver chloride electrode (auxiliary electrode), and platinum wire (counter electrode). During the sample preparation stage, an analytical balance (RADWAG, Radom, Poland) and an agate mortar were used. To reduce the influence of potential interferers, the tablet extract was filtered using a 0.22 μm Millipore filter (Burlington, MA, USA).

The Infinity Series II ultra-high performance liquid chromatography (UHPLC) with a DAD detector, an Agilent 6224 ESI/TOF mass detector (Agilent Technologies, Santa Clara, CA, USA), and an RP18 reversed-phase column Titan (Supelco, Bellefonte, PA, USA) (10 cm × 2.1 mm i.d., 1.9 µm particle size) was used to the analysis of the sample. A total of 2% of acetonitrile in water with 0.05% of formic acid (Merck reagents, Darmstadt, Germany) was used as a mobile phase at a flow rate of 0.2 mL min^−1^ [44].

### 2.2. Reagents and Solutions

Solutions with appropriate concentrations of phosphate-buffered saline (PBS) with pH = 4.5; 6.0; 6.3; 7.2; and 8.0 were prepared with Merck reagents. Selectivity studies were also performed using Merck standard solutions of Fe^3+^, Ca^2+^, Cu^2+^, Mg^2+^, Ni^2+^, Cd^2+^, Zn^2+^, Sb^3+^, Pb^2+^, NO_2_^−^, NO_3_^−^, Cl^−^, ascorbic acid, ritonavir, and lopinavir, as well as Triton X-100—Fluka (Dorset, UK). Solutions of ACV were prepared with Merck reagent. The ACV water solutions with concentrations of 5 and 1 mmol L^−1^ were prepared once every 2 weeks. On the other hand, 0.1 and 0.01 mmol L^−1^ solutions were prepared daily by dilution of the 1 mmol L^−1^ ACV standard in deionized water. All ACV standards were stored in a refrigerator and placed in an ultrasonic bath daily for a couple of minutes prior to the first use. ACV tablets were bought from a local pharmacy and wastewater samples were received from the Municipal Water Supply & Wastewater Treatment Company Ltd. (Lublin, Poland). MS grade nitric acid, formic acid, and acetonitrile were bought from Sigma-Aldrich. The water used for solution preparation was deionized using a Milli-Q system or Ultrapure Millipore Direct-Q^®^ 3UV-R (>18 MW cm).

### 2.3. Preparation of Tablets and Municipal Wastewater Samples

Tablet preparation: ACV tablets (declared value of ACV content in one tablet = 200 mg) were purchased from a local pharmacy. Three of them were carefully weighed on an analytical balance and grounded using a mortar to form a homogeneous powder. Then, the mass corresponding to the average mass of three ground tablets was weighed and quantitatively transferred to a 200 mL volumetric flask. Next, 20 mL of 1 mol L^−1^ HNO_3_ was added and the flask was filled to the mark with deionized water. Subsequently, the suspension was placed in an ultrasonic bath for 60 min. The finally obtained extract was filtered with a syringe using a 0.22 μm Millipore filter [44].

Municipal wastewater preparation: The purified sewage samples were not subjected to any additional preparation steps.

### 2.4. DPV Procedure Parameters

Differential-pulse voltammetry (DPV) was applied in ACV analysis at the BDDE in 10 mL of 0.075 mol L^−1^ PBS solution with pH = 7.2. In Table 2 the parameters of DPV technique under optimized conditions are presented. The commercially available BDDE was electrochemically cleaned in the supporting electrolyte (1.4 V for 5 s) to remove residues from the previous measurement. The background was cut out from each DPV curve and the baseline correction stage was always applied.

## 3. Results and Discussion

### 3.1. Comparison of the Electrochemical Properties of BDDE and GCE

**Figure 2:**
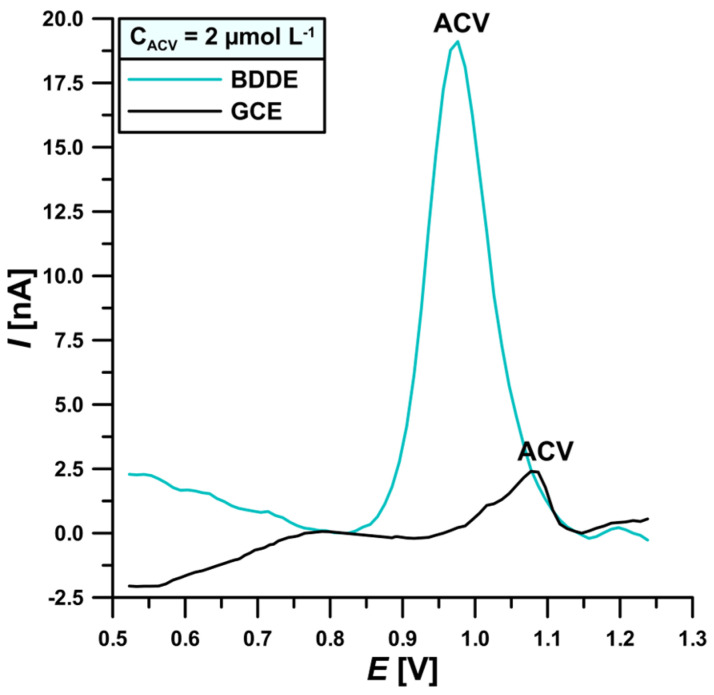
DPVs of 2 μmol L^−1^ ACV at the GCE and BDDE. Technique parameters: ΔE_A_ = 10 mV, ν = 100 mV s^−1^, and t_m_ = 10 ms.

At the beginning of the research, the quality and intensity of the analytical signal on voltammograms obtained on the GCE and the BDDE, in the presence of 2 μmol L^−1^ of ACV in 0.1 mol L^−1^ PBS pH = 7.2 solution, were compared. Figure 2 shows that the signal obtained on the BDDE is over 7.8 times higher than on the GCE, while the oxidation peak potential of ACV (*E*_p_) is shifted towards more negative potentials (0.98 V vs. 1.08 V, respectively). Additionally, the GCE peak is asymmetric and tails towards more negative potentials unlike the BDDE signal, which is symmetric, more narrowed, and close to the course of the Gaussian function. Based on the literature data, this may be related to the electrochemical properties of BDDE, including high conductivity achieved by appropriately doping diamonds with boron. It has been shown that significant changes in the conductivity of doped diamonds occur when there are 1000 carbon atoms per boron atom [45,46,47,48,49,50,51,52,53,54,55]. The commercially available BDDE was selected for further research.

**Figure 3:**
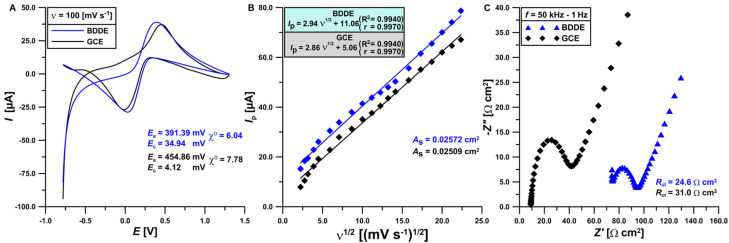
Cyclic voltammetry (CV) measurements (**A**) conducted on the GCE and BDDE; (**B**) dependence of the peak current (*I*_p_) on the square root of the scanning rate (*ν*) from 7.5 to 500 mV s^−1^; (**C**) electrochemical impedance spectroscopy (EIS) spectrum of the GCE and BDDE. All measurements were performed in a solution consisting of 0.1 mol L^−1^ KCl and 5 mmol L^−1^ K_3_[Fe(CN)_6_].

To explain the cause of the increase in the signal, the sensors were compared in a solution consisting of 5 mmol L^−1^ K_3_[Fe(CN)_6_] and 0.1 mol L^−1^ KCl using CV and EIS (Figure 3). It is worth emphasizing that such studies are available in the literature for BDDE and GCE, but not for the specific sensors described in this paper [45,46,47,48,49,50,51,52,53,54,55]. Electrooxidation and electroreduction signals of iron ions on CV voltammograms (*ν* = 100 mV s^−1^) (Figure 3A) are higher on the BDDE electrode relative to the GCE. Additionally, the relative separation of the oxidation and reduction peaks (*χ*^0^) measured for the GCE and BDDE (7.78 vs. 6.04, respectively) indicates a faster electron transfer in the case of the BDDE because (*χ*^0^ = 6.04) is closer to the theoretical value of (*χ*^0^ = 1).

Based on the results obtained for the CV voltammograms recorded for scanning rates from 7.5 to 500 mV s^−1^, the dependence of the peak current (*I*_p_) on the square root of the scan rate (*ν*) was determined for both electrodes (Figure 3B). On the basis of these relationships, the active surface areas (*A*_s_) of the GCE and BDDE were enumerated (0.02509 vs. 0.02572 cm^2^, respectively), using the Randles–Ševčík equation [56]. Thus, the BDDE was found to have a faster electron transfer and a larger active surface. In order to thoroughly examine the electrical properties of both sensors, measurements were made using EIS (50 kHz–1 Hz), and the value of charge transfer resistance (*R*_ct_) was designated for both electrodes. The impedance spectra presented in Figure 3C indicate a lower charge transfer resistance on the BDDE relative to the GCE (24.6 vs. 31.0 Ω cm^2^, respectively).

### 3.2. Optimization of the Basic Electrolyte Composition and Study of the ACV Electrode Process

**Figure 4:**
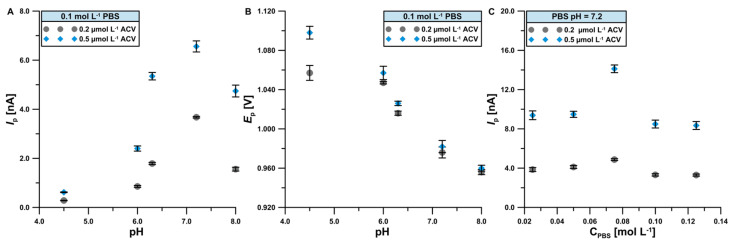
The influence of pH = (4.5; 6.0; 6.3; 7.2; and 8.0) of 0.1 mol L^−1^ PBS on 0.2 and 0.5 mol L^−1^ ACV signal (**A**) and peak potential (**B**). The influence of concentration of PBS pH = 7.2 on 0.2 and 0.5 mol L^−1^ ACV signal (**C**). DPV parameters: Δ*E*_A_ = 10 mV, *ν* = 100 mV s^−1^, and *t*_m_ = 10 ms. The standard deviation was calculated for n = 3.

As the first step, the effect of 0.1 mol L^−1^ PBS pH on the 0.2 and 0.5 μmol L^−1^ ACV signal (Figure 4A) and peak potential (Figure 4B) on the BDDE was examined. Among the tested values (pH = 4.5; 6.0; 6.3; 7.2; and 8.0), the highest peak current was obtained at pH = 7.2. The pH of the solution changes ACV acid-base balance and this is reflected in the changes in its electrochemical behavior for BDDE surface. ACV is charged at the two extremes of the pH range (pKa_1_ = 2.27 and pKa_2_ = 9.25). Consequently, the molecule bears a positive charge in solutions up to pH = 2.27. Then, when the pH increases the formation of neutral and anionic species of ACV is favored and this could increase the interaction with the BDDE surface which affects the charge transfer. Finally, at pH = 8, electrooxidation responses decrease due to the predominance of negatively charged species of ACV [17]. According to Figure 4B, the peak potentials of ACV move towards lower values as pH increases, which indicates that protons are included in the oxidation reaction of ACV on the BDDE [57]. Next, the effect of the PBS concentration ranging from 0.025 to 0.125 mol L^−1^ on the ACV signal was examined (Figure 4C). Ultimately, the best results were achieved in 0.075 mol L^−1^ PBS pH = 7.2. The increase in ACV signal is related to the improvement of the supporting electrolyte’s electrical conductivity. At concentrations higher than 0.075 mol L^−1^, subsequent processes may occur that contribute to a reduction in the efficiency of ACV determination.

**Figure 5:**
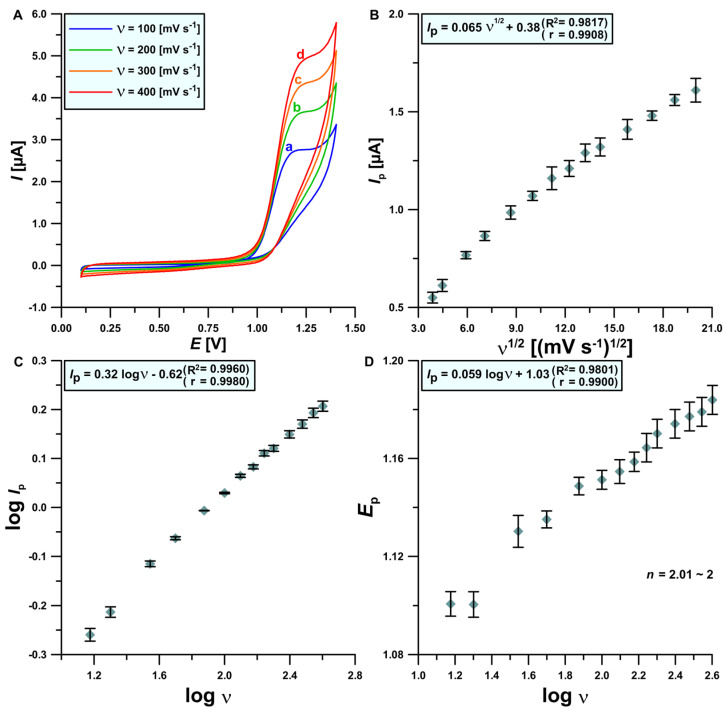
(**A**) CVs obtained on the BDDE in the optimized electrolyte formulation; (**B**) relationship between the peak current (*I*_p_) and the square root of the scanning rate (*ν*)–(*ν*: 15–400 mV s^−1^); (**C**) log *I*_p_ and log *ν* dependence; (**D**) E_p_ and log *ν* dependence. The standard deviation was calculated for n = 3.

**Figure 6:**
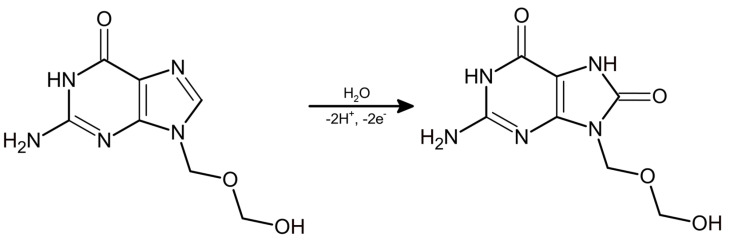
ACV oxidation mechanism.

In the next stage of our research, CV voltammograms were recorded in the optimized electrolyte composition with the presence of 50 μmol L^−1^ ACV. The influence of changes in the scanning rate ranging from 15 to 400 mV s^−1^ on the ACV signal was checked. It is noticeable that as the scanning speed increases towards higher values, the ACV electrooxidation peak current also increases significantly (Figure 5A). In addition, the ACV signal shift towards more positive values of potential is visible, along with an increase in the scanning rate. The lack of the reduction peak is proof of the irreversibility of the tested electrode process [56]. The linear relationship between *I*_p_ and *ν*^1/2^ (Figure 5B) in combination with the log *I*_p_ and log *ν* (Figure 5C) dependence (the slope of the curve approaches the theoretical value of 0.5) is proof of the purely diffusive nature of the studied process. Based on the slope of the curve obtained from the dependency between *E*_p_ and log *ν* (Figure 5D) and the Laviron equation [58], the *n* value (electron transfer number involved in the rate-determining step) was determined. The received value (*n* = 2.01) means that two electrons take part in the electrooxidation reaction of ACV on the BDDE. Moreover, oxidation of ACV on BDDE involves a deprotonation step and breaking of the double bond in the imidazole ring between the N(7) = C(8) atoms. Ultimately, an oxoguanine analog is obtained as the final product of this reaction [2,17,19] (Figure 6).

### 3.3. Procedure Optimization

It was found that the investigated process is purely diffusive (Figure 5). To facilitate access of ACV molecules to the electrode surface, the influence of the base electrolyte solution mixing time (*t*) before DPV registration on the ACV (0.2 and 0.5 μmol L^−1^) analytical signal was first checked. Measurements were carried out in the t ranging from 5 s to 30 s. Among the tested values, the highest signal was achieved for the mixing time of 5 s, which is consistent with the nature of the tested process. As t increased, the current intensity of the ACV peak decreased and at *t* > 15 s, a plateau was established.

**Figure 7:**
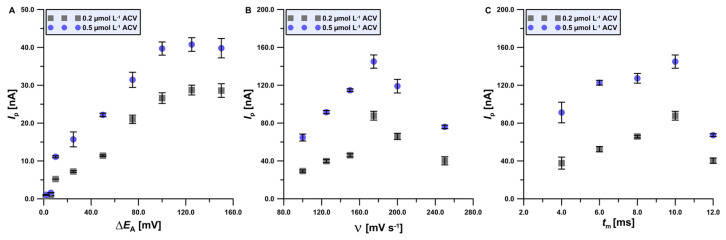
The dependence in the presence of 0.2 and 0.5 mol L^−1^ ACV in the optimized electrolyte formulation between (**A**) *I*_p_ and Δ*E*_A_; (**B**) *I*_p_ and *ν*; (**C**) *I*_p_ and *t*_m_. The standard deviation was calculated for n = 3.

In the next part of the research, the DPV technique parameters such as amplitude (Δ*E*_A_), scan rate (*ν*), and modulation time (*t*_m_) were optimized (Figure 7). When selecting the optimal value of the tested parameters, the height of the analytical signal, its shape, and signal repeatability were considered. First, ΔE_A_ was changed from 2 to 150 mV, with constant values of *ν* = 100 mV s^−1^ and *t*_m_ = 10 ms. The highest signal from the two ACV additions (0.2 and 0.5 μmol L^−1^) was obtained for Δ*E*_A_ = 125 mV. Then, the scanning speed value was optimized by changing it from 100 to 250 mV s^−1^, with constant Δ*E*_A_ = 125 mV and *t*_m_ = 10 ms. The *ν* = 175 mV s^−1^ value was chosen as optimal. Finally, the influence of modulation time changes in the range from 4 to 12 ms was checked similarly and *t*_m_ = 10 ms was the most optimal value.

During the next stage of this research, the repeatability of the 0.5 μmol L^−1^ ACV signals with optimized DPV technique parameters on the BDDE electrode was examined. The relative standard deviation (RSD) was checked for 10 measurements. The obtained results allowed determining that RSD = 10.2%. To improve signal repeatability, the effect of applying different purification potentials while mixing the solution for a certain period before the measurement was tested. It was found (Table 3) that applying a potential of 1.4 V for 10 s causes a significant improvement in the value of the obtained RSD (%). However, the best results were achieved by applying a potential of 1.4 V during the already optimized value of the mixing time of the basic electrolyte solution (*t* = 5 s).

### 3.4. Analytical Parameters of the Procedure and the Influence of Interference Species

**Figure 8:**
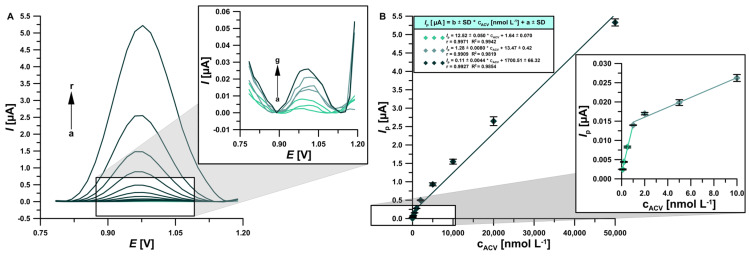
**(A**) DPVs registered on the BDDE in 0.075 mol L^−1^ PBS pH = 7.2 in the presence of a rising ACV concentration: (a→r, 0.0001, 0.0002, 0.0005, 0.001, 0.002, 0.005, 0.01, 0.02, 0.05, 0.1, 0.2, 0.5, 1.0, 2.0, 5.0, 10.0, 20.0, and 50.0 μmol L^−1^). (**B**) The linear calibration curve. All measurements were performed under optimized DPV parameters. The standard deviation was calculated for n = 3.

To determine the quality and capabilities of the newly developed procedure, the linearity ranges of the calibration curve and the limit of detection (LOD) and quantification (LOQ) were determined under optimized conditions of the base electrolyte composition and DPV parameters. For this purpose, DPV signals were recorded for increasing ACV concentrations (0.1–50,000 nmol L^−1^) (Figure 8A). On their basis, a calibration curve (Figure 8B) was determined, which consists of three linearity ranges: 0.0001–0.001 μmol L^−1^ (r = 0.9971, sensitivity = 12.52 ± 0.050 μA/nmol L^−1^), 0.001–0.01 μmol L^−1^ (r = 0.9909), and 0.01–50.0 μmol L^−1^ (r = 0.9927) (0.1–1.0 nmol L^−1^; 1.0–10.0 nmol L^−1^ and 10.0–50,000 nmol L^−1^). The LOD and LOQ were calculated from the following equations: 3SD_a_/b and 10SD_a_/b (SD_a_ is the standard deviation of intercept for n = 3 and b is the slope of the calibration plot), respectively [59]. The LOD value was determined at 0.0299 nmol L^−1^ and LOQ = 0.0995 nmol L^−1^. RSD between measurements for one standard addition over the entire range of the calibration curve ranged between 0.93% and 4.40%. This result proves very good signal repeatability. It is worth emphasizing that the developed ACV trace-level determination procedure is characterized by the lowest detection limit of all voltammetric procedures described in the literature (Table 1). This is the first time that a bare electrode outperforms modified ones in ACV analysis.

Selectivity tests were performed in the presence of 0.1 μmol L^−1^ ACV and a 10-fold excess of ions such as Fe^3+^, Ca^2+^, Cu^2+^, Mg^2+^, Ni^2+^, Cd^2+^, Zn^2+^, Sb^3+^, Pb^2+^, NO_2_^−^, NO_3_^−^, Cl^−^, and ascorbic acid, ritonavir, lopinavir as well as Triton X-100 (0.2–2.0 ppm). The presence of these substances did not affect the ACV analytical signal by more than ± 10%.

### 3.5. Sample Analysis

**Figure 9:**
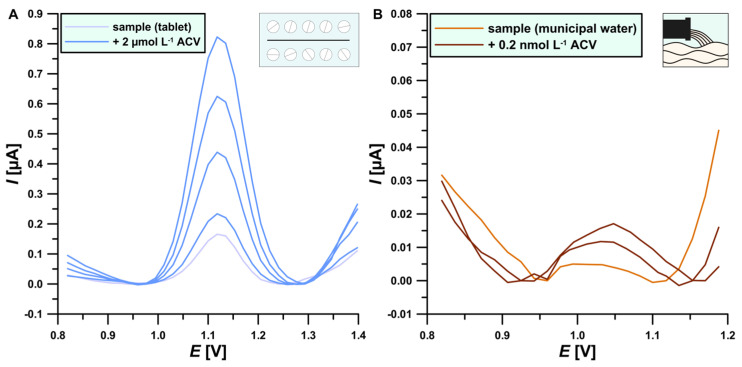
DPVs recorded on the BDDE in the presence of the tested sample: (**A**) tablet extract; (**B**) purified municipal wastewater and the tested sample + the addition of the ACV standard.

The samples were analyzed using the standard addition method (voltammetry and chromatography) as well as external calibration (chromatography). The comparative methods are UHPLC-DAD and UHPLC-MS (TOF). Quantification of DPV tablets was performed by adding 1 μL of the extract to the electrochemical cell (Figure 9A). The analysis of wastewater samples was carried out by diluting the starting solution 10 times to reduce matrix effects (Figure 9B). The results of ACV determinations in the tablet extract and purified municipal wastewater performed using the voltammetry technique as well as chromatography reference methods are presented in Table 4.

**Figure 10:**
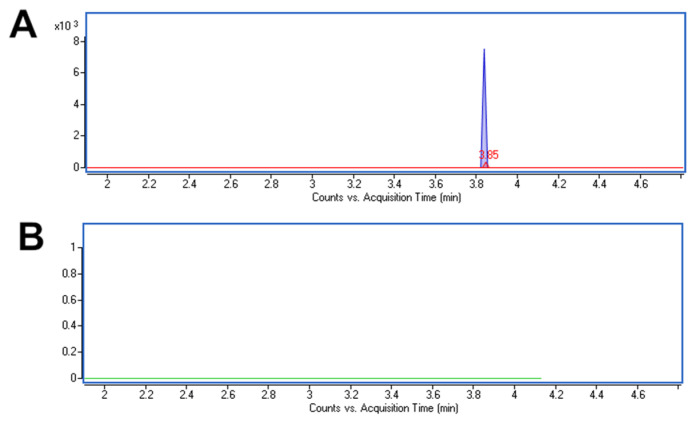
UHPLC-MS (TOF) analysis of ACV: (**A**) EIC in the mass range of *m*/*z*: 226.092–226.094 for ACV standard at a concentration of 0.1 μmol L^−1^ (blue line) and the sewage sample (red line); (**B**) EIC in the mass range of *m*/*z* 226.092–226.094 for deionized water (blanc).

In the case of the analysis of the tablet extract, consistent results were obtained both using the DPV procedure and chromatographic reference methods [14,15,16]. The obtained recovery values are close to 100% (in the range of 94.47–96.26%), indicating a low effect of the sample matrix on the ACV peak. During the analysis of wastewater samples, the advantages of the voltammetric methods emerged. In the case of the DPV procedure, the ACV concentration in a wastewater sample was determined at 1.34 nmol L^−1^, which is a value consistent with the literature data [9]. In the case of the reference methods, it was not possible to perform a quantitative analysis of not spiked samples. Only UHPLC-MS (TOF)) was able to detect ACV in the sample (Figure 10).

The voltammetric and chromatographic methods allowed for the determination of ACV in the enriched sample (wastewater samples spiked with 100 ng L^−1^ ACV standard solution). The recovery values calculated for the spiked samples are in the range of 92.27–95.21%. The recovery analysis showed a low impact of matrix effects on the ACV signal.

## 4. Conclusions

For the first time, unmodified BDDE combined with DPV was used for the determination of ACV. The developed procedure using BDDE allows for achieving the lowest detection limit (LOD = 0.0299 nmol L^−1^) of all the voltammetric ACV determination procedures described in the literature. Additionally, the developed procedure has a low limit of quantification (LOQ = 0.0995 nmol L^−1^) as well as a wide range of linearity of the calibration curve: 0.0001–0.001 μmol L^−1^; 0.001–0.01 μmol L^−1^; and 0.01–50.0 μmol L^−1^. Our sensor is unmodified, which significantly shortens the preparation time compared to complex sensors described in the literature. The time needed from the beginning of the measurement to obtain the voltammogram is also very short (the total time of one measurement is less than 18 s). Moreover, the proposed procedure with BDDE was successfully used to analyze pharmaceutical and municipal wastewater samples. The obtained results are consistent with measurements made using chromatographic methods. The lack of time-consuming preparation of the sensor and the low consumption of samples and reagents meet the requirements of green chemistry. The results are promising for the potential application of the BDDE in simple, fast, and sensitive ACV quantification.

## Figures and Tables

**Table 1:** ACV determination procedures.

Technique (Sensor)	Linear Range (µmol L^−1^)	LOD (µmol L^−1^)	Matrix Type	Ref.
**Spectroscopic Methods**				
PLS—NIRS	–	0.22–9.55	plasma samples	[10]
Spectrophotometry	497.31–7193.29	–	pharmaceutical samples	[11]
Spectrophotometry	8.88–35.52	1.13	pharmaceutical samples	[12]
Spectrofluorimetry	1.13–11.10	0.09	pharmaceutical samples	[12]
FI–CL	0.89–355.22	0.27	pharmaceutical samples	[13]
**Chromatographic Methods**				
HPLC–UV	–	0.018	plasma samples	[14]
UHPLC–HESI–MS/MS	0.0044–8.88	0.0022	plasma samples	[15]
LC–ESI–MS	0.022–0.22	0.0044	aqueous humor	[16]
**Voltammetric Methods**				
LCAdSV (MWNTs-DHP film-coated GCE)	0.08–10.0	0.03	tablets	[8]
DPAdSV (C_60_/GCE)	0.09–6.0	0.0148	pharmaceutical samples, human urine, and blood plasma	[1]
LCAdSV (GCE/TFM)	0.09–0.53	0.001	synthetic sample that contains antiretroviral drugs or ATP and DNA	[7]
SWV (FTO)	4.0–40.0	1.25	pharmaceutical samples	[17]
DPAdSV (Cysteic acid (DES)/nano-NaOH/GCE)	0.03–0.1 0.1–3.2	0.008	tablets and biological fluids	[6]
DPV (UTGE and GCE)	(UTGE) 4–70 (GCE) 2–100	(UTGE) 1.0 (GCE) 0.35	spiked human urine	[5]
DPV (PEBT/GCE)	0.03–0.3 0.3–1.5	0.012	human blood serum, pharmaceutical formulations	[18]
LSAdSV (OPPY/CNT/GCE)	0.03–10.0	0.01	pharmaceutical and clinical preparations	[2]
DPV (PGE)	1.0–100.0	0.3	pharmaceutical formulations	[19]
SWAdSV (NC/GPE)	0.05–1.0	0.0002	pharmaceutical and biological samples	[20]
SWV (GC/OPPy/Acy)	0.5–10.0	0.2	pharmaceutical samples	[21]
LSAdSV (FeMoO_4_-GO/GCE)	0.1–10.0 10.0–100	0.02	drug samples	[22]
DPV (β-CD/TiO_2_ NPs/CPE)	0.09–2.98 2.98–47.61	0.021	blood serum samples	[23]
LSAdSV (rGO–TiO_2_–Au/GCE)	1.0–100	0.3	tablet samples	[24]
SWV (MBZ/TMHPP Cu(II)-modified GE)	0.01–1000.0	0.01	pharmaceutical formulations and urine samples	[25]
SWAdSV (β-CD/EPPGE)	0.05–0.6 1.0–9.0	0.00759	tablets, human urine	[26]
DPV (CdO/Fe_3_O_4_/CPE)	1.0–100.0	0.3	tablets, blood serum, and urine samples	[27]
DPAASV (Ag NPs/CdS NWs/RG/GCE)	0.01–4.0 4.0–40.0	0.0033	blood serum, tablet, and topical cream samples	[28]
DPAASV (CS-MWCNTs+TiO_2_ NPs/PCC/nanoporous GCE)	0.03–1.0	0.01	human fluid and tablet samples	[29]
DPV (P-OAP/MWCNTs-ZnO NPs-CPE)	0.399–35.36	0.3	pharmaceutical formulations	[30]
SWAdSV (EPPEG)	0.5–3.0	0.0607	pharmaceutical formulations and human plasma	[31]
LSV (AuNPs/CNTs-COOH/GSE)	0.192–52.0 52.0–200.0	0.057	pharmaceutical preparations	[32]
SWV (ND@Dy_2_O_3_-IL/CPE)	0.097–116.6	0.029	human serum sample	[33]
DPV (p-ABSA-GCE)	0.2–9.0	0.0557	tablets	[34]
SWV (SWNT/Naf/GCE)	0.01–30.0	0.0018	human urine sample	[35]
CV (polyGly-GO_red_-GCE)	5.0–5000.0	–	pharmaceutical preparations	[36]
DPV (rGO/Pd@PACP/PGE)	0.1–0.5	0.0513	–	[37]
SWV (CGMDE)	0.2–2.0	0.07	–	[38]
DPAdSV (RuNPs/TBA/PGE)	0.003–0.030 0.030–3.0	0.0008	tablet and urine samples	[39]
DPV (GC/CNT/ILC/RGO/MnO_2_)	0.01–30.0	0.000843	human serum	[40]
SWV (γ-Fe_2_O_3_-Bent/CPE)	0.5–8.0	0.00155	pharmaceutical and urine samples	[43]
DPAdSV (aSPCE)	0.0005–0.05 0.005–1.0	0.00012	tablets	[44]
**DPV (BDDE)**	**0.0001–0.001** **0.001–0.01** **0.01–50.0**	**0.0000299**	**municipal water samples and tablets**	**This work**

**Techniques:** PLS–NIRS—partial least squares regression model coupled with near-infrared spectroscopy; FI–CL—flow-injection analysis–chemiluminescence detection; HPLC–UV—high-performance liquid chromatography–ultraviolet detection; UHPLC-HESI–MS/M—ultra-high-performance liquid chromatography–heated electrospray ionization–tandem mass spectrometry; LC–ESI–MS—liquid chromatography–electrospray ionization–mass spectrometry; LCAdSV—linear cyclic adsorptive stripping voltammetry; DPAdSV—differential-pulse adsorptive stripping voltammetry; SWV—square-wave voltammetry; DPV—differential-pulse voltammetry; SWAdS—square-wave adsorptive stripping voltammetry, LSAdSV—linear sweep adsorptive stripping voltammetry; DPAASV—differential-pulse adsorptive anodic stripping voltammetry; LSV—linear sweep voltammetry; CV—cyclic voltammetry. **Electrodes:** MWNTs-DHP film-coated GCE—multi-wall carbon nanotubes (MWNTs)-dihexadecyl hydrogen phosphate (DHP) film-coated glassy carbon electrode (GCE); C_60_/GCE—fullerene- C_60_-modified glassy carbon electrode; GCE/TFM—glassy carbon electrode modified with thin mercury film; FTO—fluoride doped oxide electrodes; cysteic acid (DES)/nano-NaOH/GCE)—glassy carbon electrode modified with nanorods of NaOH decorated with polymeric film in the presence of deep eutectic solvent; UTGE—ultra-trace graphite electrode; GCE—glassy carbon electrode; PEBT/GCE—glassy carbon electrode modified with coated with polymerized eriochrome black T; OPPY/CNT/GCE—glassy carbon electrode modified with a bilayer of multi-walled carbon nanotube/tiron-doped polypyrrole; PGE—pencil graphite electrode; NC/GPE—nanoclay modified graphite paste electrode; GC/OPPy/Acy—glassy carbon electrode coated with molecularly imprinted, overoxidized polypyrrole (OPPy) dotted with acyclovir; FeMoO_4_-GO/GCE—ferrous molybdate nanorods and graphene oxide composited glassy carbon electrode; β-CD/TiO_2_ NPs/CPE—carbon paste electrode modified with titanium (IV) oxide nanoparticles and coated with β-Cyclodextrin; rGO–TiO_2_–Au/GCE—reduced graphene oxide–TiO_2_–Au nanocomposite-modified glassy carbon electrode; MBZ/TMHPP Cu (II)-modified GE—gold electrode modified with 2-mercaptobenzothiazole–[5,10,15,20-tetrakis-(3-methoxy-4-hydroxyphenyl)porphyrinato]copper(II); β-CD/EPPGE—electropretrated pencil graphite electrode modified with polymerized β-Cyclodextrin; CdO/Fe_3_O_4_/CPE—carbon paste electrode modified with magnetic cadmium oxide CdO/Fe_3_O_4_; Ag NPs/CdS NWs/RG/GCE—glassy carbon electrode modified with silver nanoparticles/cadmium sulfide nanowires/reduced graphene oxide nanocomposite; CS-MWCNTs+TiO_2_ NPs/PCC/nanoporous GCE—nanoporous glassy carbon electrode modified with polymeric film decorated multi-walled carbon nanotubes and TiO_2_ nanoparticles; P-OAP/MWCN—Ts-ZnO NPs-CPE—poly (o-aminophenol)/multi-walled carbon nanotubes-ZnO nanoparticles-carbon paste electrode; EPPEG—electropretreated pencil graphite electrode; AuNPs/CNTs-COOH/GSE—graphite sheet electrode modified with carboxylatedcarbon nanotubes and Au nanoparticles; ND@Dy_2_O_3_-IL/CPE—nanodiamond decorated dysprosium oxide and ionic liquid (IL) modified carbon paste electrode; p-ABSA-GCE—glassy carbon electrode modified with p-aminobenzene sulfonic acid; SWNT/Naf/GCE—glassy carbon electrode modified with single-walled carbon nanotubes and nafion composite film; polyGly-GO_red_–GCE—glassy carbon electrode modified with reduced graphene oxide–polyglycine film composite; rGO/Pd@PACP/PGE—pencil graphite electrode modified with reduced graphene oxide/palladium nanoparticles/poly(2-amino-4-chlorophenol) (rGO/Pd@PACP) nanocomposite; CGMDE—controlled growth mercury drop electrode; RuNPs/TBA/PGE—pencil graphite electrode modified by ruthenium nanoparticles and thiobarbituric acid; GC/CNT/ILC/RGO/MnO_2_—glassy carbon electrode modified with layers of multi-walled carbon nanotubes (CNTs), an ionic liquid crystal (ILC), graphene (RGO) and MnO_2_; γ-Fe_2_O_3_-Bent/CPE—carbon paste electrode modified with nano γ-Fe_2_O_3_ composite and bentonite clay; aSPCE—electrochemically activated screen-printed carbon electrode; BDDE—boron-doped diamond electrode.

**Table 2:** DPV parameters under optimized conditions.

Parameter	Value
Electrochemical cleaning step	1.4 V (5 s)
Analytical signal recording range	from 0.1 V to 1.4 V
Scan rate (*ν*)	175 mV s^−1^
Amplitude (Δ*E*_A_)	125 mV
Modulation time (*t*_m_)	10 ms
Equilibrium time	5 s

**Table 3:** Effect of the electrochemical purification step on the ACV signal.

Electrochemical Cleaning Step	RSD (%)
-	10.2
1.2 V (10 s)	13.1
1.4 V (10 s)	6.64
1.4 V (5 s)	3.99

**Table 4:** Results of ACV determination in real samples.

Parameter	Measurement Method		
	DPV LOD: 0.0299 nmol L^−1^ LOQ: 0.0995 nmol L^−1^	UHPLC-DAD LOD: 32 nmol L^−1^	UHPLC-MS (TOF) LOD: 1.1 nmol L^−1^
	Tablet (Declared Value = 200 mg)		
**Found ± SD** **(n = 3)**	188.93 ± 9.45 mg	192.51 ± 5.2 mg	191.92 ± 5.7 mg
**Recovery ± Coefficient of variation**	94.47 ± 5.00%	96.26 ± 2.58%	95.96 ± 2.97%
**Parameter**	**Purified municipal wastewater**		
**Found ± SD** **(n = 3)**	1.34 ± 0.0659 nmol L^−1^	>LOD >LOQ	Detected >LOQ
Recovery * ± Coefficient of variation	92.27 ± 2.59%	93.42 ± 8.10%	95.21 ± 8.61%

**Recovery [%]** = (found × 100)/declared value; **Coefficient of variation [%]** = (SD × 100)/found; * ACV = 100 ng L^−1^ standard addition.

## Data Availability

The original contributions presented in the study are included in the article, further inquiries can be directed to the corresponding author.
